# Endodontic access cavity preparation: challenges and recent advancements

**DOI:** 10.1038/s41415-025-8442-8

**Published:** 2025-04-11

**Authors:** Ahmed Elmatary, Emad Moawad, Omid Heidarifar, Simon Stone

**Affiliations:** 168390524092503365280 School of Dentistry, Institute of Life Courses and Medical Sciences, University of Liverpool, Liverpool, UK; Liverpool University Dental Hospital NHS Foundation Trust, Liverpool, United Kingdom; 177721721838009553213 School of Dental Sciences, Newcastle University, Newcastle upon Tyne, UK; The Newcastle upon Tyne Hospitals NHS Foundation Trust, Newcastle upon Tyne, United Kingdom

## Abstract

Odontogenic pain from various dental issues significantly impacts quality of life, necessitating effective treatment during emergency dental care. Endodontic access cavity preparation is essential for alleviating symptoms and preventing further infection. This procedure aims to achieve symptom relief while conserving as much tooth structure as possible. This paper discusses the importance of proper endodontic access, emphasising the balance between adequate disinfection and preserving tooth integrity. It also identifies challenges in endodontic access cavity preparation. This study reviews existing literature on endodontic access, examining different approaches to access cavity preparation and the tools and techniques used. Factors affecting access difficulty, including tooth anatomy, patient-related challenges and operator skills, are evaluated, alongside advancements in imaging and instrumentation. The review shows conservative techniques, like minimally invasive access cavities, which helps to preserve tooth structure but requires advanced skills and may risk incomplete disinfection. Cone beam computed tomography (CBCT) aids in complex cases, improving canal location accuracy. Operator skills and proper equipment are key to success. Traditional cavities offer better access but can weaken the tooth, while conservative approaches maintain structure but demand more expertise. Imaging tools, including CBCT, are beneficial for complex anatomy but can be costly. To conclude, effective endodontic access cavity preparation requires a balanced approach tailored to each case. While conservative methods offer advantages in preserving tooth structure, their success depends on operator expertise and equipment. Incorporating imaging advancements like CBCT can enhance access success, especially in anatomically complex cases, but careful assessment of case complexity remains crucial.

## Key points

- Provides an improved understanding of endodontic emergency management.
- Offers an insight into endodontic access cavity procedures.
- Increases awareness of conservative endodontic access cavity approaches, the challenges and complications in endodontic access cavity procedures, and the technological advancements used in the endodontic access cavity.

## Introduction

Odontogenic pain arising from pulpitis, pulpal necrosis, periradicular periodontitis or dental abscess can significantly and substantially impact quality of life.^1^ Treatment at ‘unscheduled dental care' appointments varies significantly because of time constraints; this is particularly challenging where there is uncertainty about the origin of pain and potential for spread of infection (including sepsis), and therefore, there is a need to investigate, rapidly diagnose, consent and carry out operative treatment to provide symptomatic relief. Priority should be given to pain relief and drainage of pus in the case of acute dental abscess and, where teeth are deemed restorable, plan for follow-up care. These management strategies are designed to address endodontic issues effectively and enable teeth to be retained that are pain-free and functional.

Endodontic access cavity preparation is often considered the initial stage of endodontic treatment. Conservative treatment with removal of some or all the coronal pulp may be considered where there is vital but inflamed pulp remaining (pulp cap, partial or full pulpotomy with calcium silicate cements), allowing comprehensive care, including restoration, in one visit.^2^^,^^3^^,^^4^ Perhaps more commonly, an access cavity preparation is created along with coronal pulpotomy or total pulpectomy and temporisation, allowing root canal therapy to be carried out at a later appointment. Where dental trauma has occurred, the relevant guidelines should be consulted and followed concerning the need and timing of root canal treatment.^5^

**Fig. 1:**
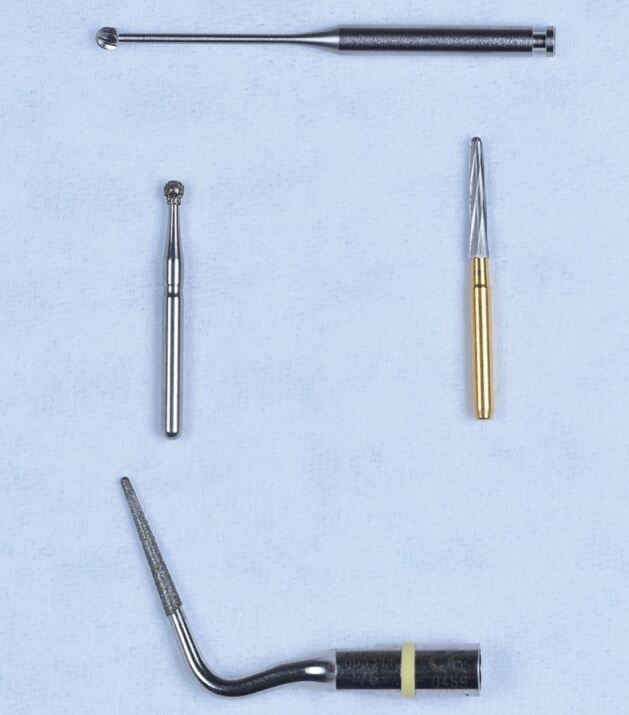
End-cutting round diamond bur, often used for endodontic access followed by a non-end-cutting tungsten carbide Endo-Z bur (Dentsply Sirona, USA), often used for deroofing of the pulp chamber. A Meisinger bur (Hager & Meisinger, Germany) is also shown and can be used for refining the access cavity and troughing to identify canal orifices (note the long shank which can aid operator visibility during access). A diamond-coated ET18D ultrasonic tip (Acteon, UK) is also shown and can be used for this purpose also

The cornerstone of endodontic treatment is predictably accessing the coronal part of the pulp to identify the main root canal anatomy and commence bio-mechanical disinfection of the root canal system. This is carried out using a combination of end-cutting and non-end-cutting burs (Fig. 1), with end-cutting burs being used to cut through enamel, dentine and restorative materials and entering the coronal pulp chamber before switching to non-end-cutting burs for pulp chamber unroofing.

**Fig. 2:**
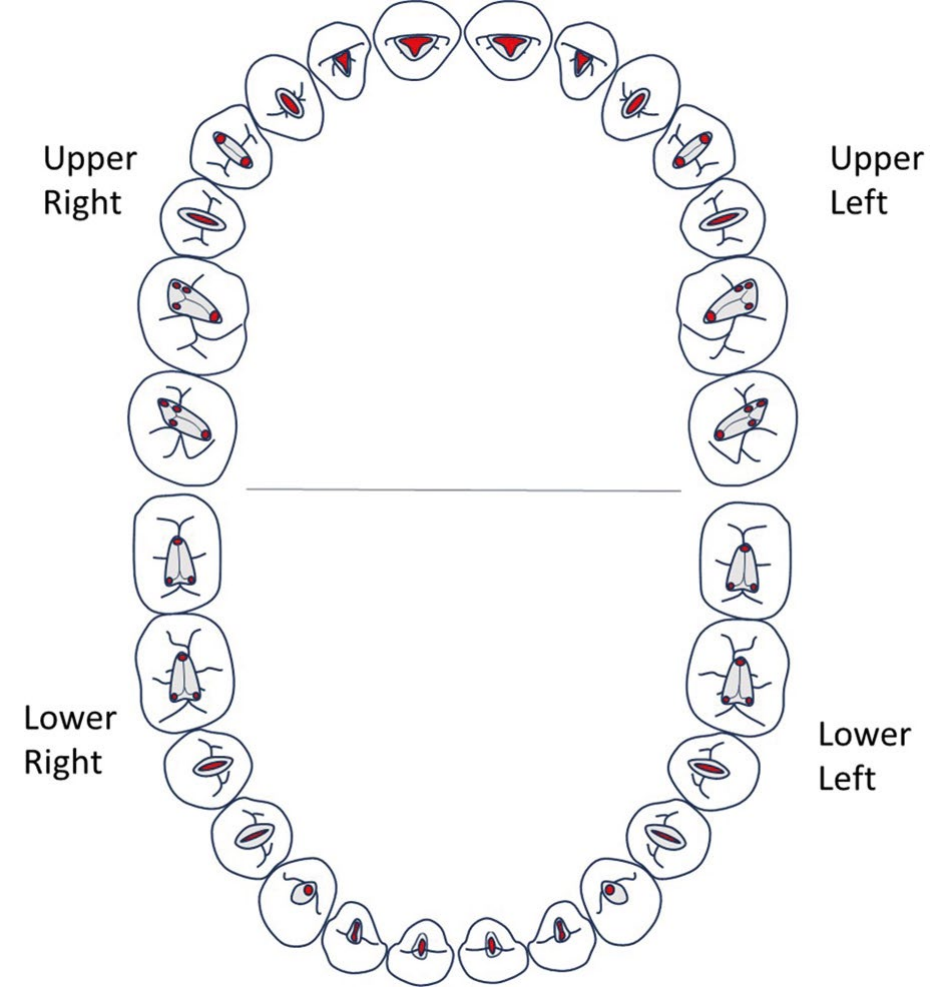
Classical access cavity shapes for upper and lower adult dentition. These can be modified to accommodate variations in anatomy identified on scouting of canals or from pre-operative imaging, such as periapicals or CBCT

The principle objective of access cavity preparation is to remove the roof of the pulp chamber, locate the canal orifice(s), and establish straight-line access. This enables subsequent steps, including coronal flaring, creating a reproducible glide path, determining the working length, and carrying out biomechanical preparation and disinfection of the root canal system. Ultimately, the procedure aims to achieve effective obturation, ensure the longevity of the restoration, and preserve as much of the natural tooth structure as possible.^6^ This can only be achieved through knowledge of the underlying dental anatomy (Fig. 2) and by selecting the most appropriate bur or burs to use. The depth of an access cavity preparation may vary depending on the depth of the coronal pulp horn, which in older, worn, heavily carious or restored teeth may have receded and been replaced by secondary or tertiary dentine deposition, necessitating a careful approach to canal location.

Vision is often a limiting factor and magnification, particularly with an attached light along with standard operating light, will significantly improve the ability to correctly identify canal orifices and be conservative of coronal dentine. The dental operating microscope offers unrivalled vision in this respect regarding the clinical ability to visualise anatomical challenges, such as sclerosed canal entrances and variations, such as additional root canals. A well-made access cavity will improve the ability to disinfect and instrument the canal system and significantly reduce the risk of iatrogenic errors.

**Fig. 3:**
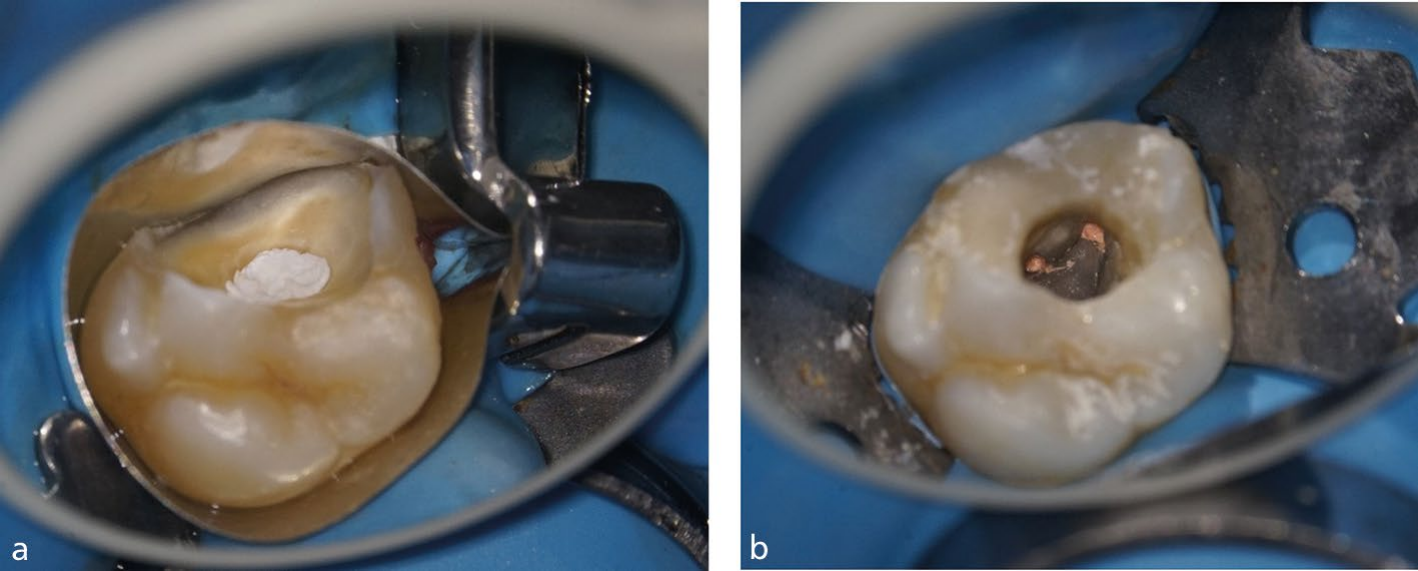
a) The 26 requiring rewalling to aid isolation and containment of irrigant. A Greater Curve matrix band was used in this case and the pulp chamber was blocked out by polytetrafluoroethylene. b) The 26 was rewalled with Fill-Up! dual cure bulk fill composite (Coltene, Switzerland). Image courtesy of Dr Shakil Umerji

What is often forgotten, particularly for patients returning following emergency treatment, is the importance of stripping down teeth and assessing their restorability. Both Beach *et al*. (1996) and Krakow *et al*. (1977) identified microleakage following initial endodontic temporisation and it is important that teeth can be adequately isolated to facilitate disinfection.^7^^,^^8^^,^^9^ Defective restorations and complete caries removal should be undertaken as part of the access cavity preparation to confirm restorability. Restorability can be influenced by a multitude of factors, including whether a successful coronal seal could be achieved following endodontic therapy, as well as the presence of a ferrule (1.5-2 mm of supragingival sound tooth structure).^10^ It is worth remembering that teeth scheduled for endodontic treatment have often been subject to a lifetime of caries, restorations, trauma or tooth wear and as such, the original anatomy of the crown may have been altered significantly. Accessing teeth through crowns is often challenging and increases case complexity,^11^ increasing the risk of iatrogenic damage and the likelihood of root perforation. The recommendation is therefore to strip down teeth and make decisions about restorability early, with thought given to what the final restoration will look like.^12^ This involves the removal of poorly adapted crowns and other restorations before access preparation. Once restorability is confirmed, a tooth can be re-walled efficiently, thus aiding isolation and containment of irritants (Fig. 3).

It is often emphasised that achieving the right balance in endodontic access is crucial. This balance involves preserving as much healthy tooth structure as possible while effectively removing coronal interferences, necrotic pulp tissue, and providing straight line access to the canal orifices. There is nothing inherently wrong with the traditional access cavity shapes (Fig. 2); however, advances in magnification, with many practitioners using dental loupes, microscopes and improved lighting, have led to a trend towards more conservative access cavities. Recent social media trends have popularised minimal access cavities and innovative designs like ‘ninja' and ‘truss' cavities, which aim to further preserve sound tooth tissue. This paper will aim to discuss the merits and pitfalls of these access cavities, as well as common challenges encountered during endodontic access, complications and errors.

## Key factors in access preparation

Access cavity preparation in endodontics presents several challenges.

### Tooth factors

There is significant variation in tooth anatomy which can complicate access cavity preparation. Crown morphology, including the shape and size of the crown, and the presence or absence of direct and indirect restorations may make pulp chamber and root canal identification more challenging. Pulp chambers and canal orifices can be obstructed by pulp stones, calcification, or cutting debris from access cavity preparation, complicating their identification. Accessing through indirect restorations can result in the excessive removal of restorative material and dentine, impacting the integrity of the restoration and potentially causing fractures, chipping, or dislodgement of the restoration.^13^ The position of the tooth within the arch also plays a role; posterior teeth can present difficulties in access and visibility.^14^ Tooth inclination and rotation can lead to loss of orientation and increase the risk of perforation. Some teeth, such as those in patients with dentinogenseis imperfecta, have poor-quality dentine, short clinical crowns and often obliterated root canal anatomy, making identification of the original root canal very difficult.

### Patient factors

Patient-related factors can complicate access cavity preparation. Physical limitations, such as an inability to recline in the chair, can restrict access and visibility. Anxiety and a strong gag reflex, particularly in posterior teeth, can further complicate the procedure. Additionally, reduced mouth opening (<25 mm) can increase treatment difficulty by limiting access and visibility and increasing stress on instruments.^15^

### Operator factors

Limited experience and misjudgement in evaluating case complexity, alongside inadequate use of essential tools, such as magnification, can impede successful canal location and disinfection. However, effective training and investment in appropriate equipment can be costly and require significant financial resources.

## Guidance for achieving good outcomes

**Fig. 4:**
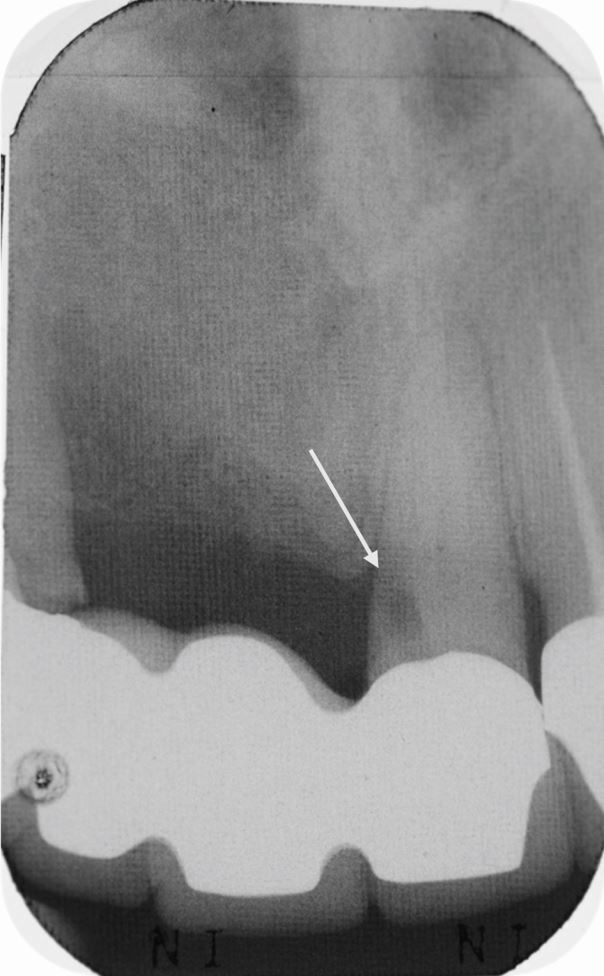
The 21 became non-vital underneath a four-unit bridge which has removed all the normal anatomical landmarks. The access cavity had been misaligned and, in this case, resulted in iatrogenic perforation of the root. Note the white arrow showing the location of the perforation

A thorough pre-operative assessment is necessary to identify case difficulty and potential pitfalls. Teeth requiring endodontic treatment can be heavily restored with either intra-coronal or extra-coronal restorations, with dental caries and cracks also being commonly present.^9^ In these cases, dismantling of the intra- or extra-coronal restoration is recommended to fully assess restorability and reduce the risk of unnecessary sound tooth tissue removal or perforation caused by a loss of anatomical landmarks associated with the tooth.^9^ Figure 4 shows an iatrogenic perforation occurring as a result of a misaligned access through an abutment tooth of a four-unit bridge.

**Fig. 5:**
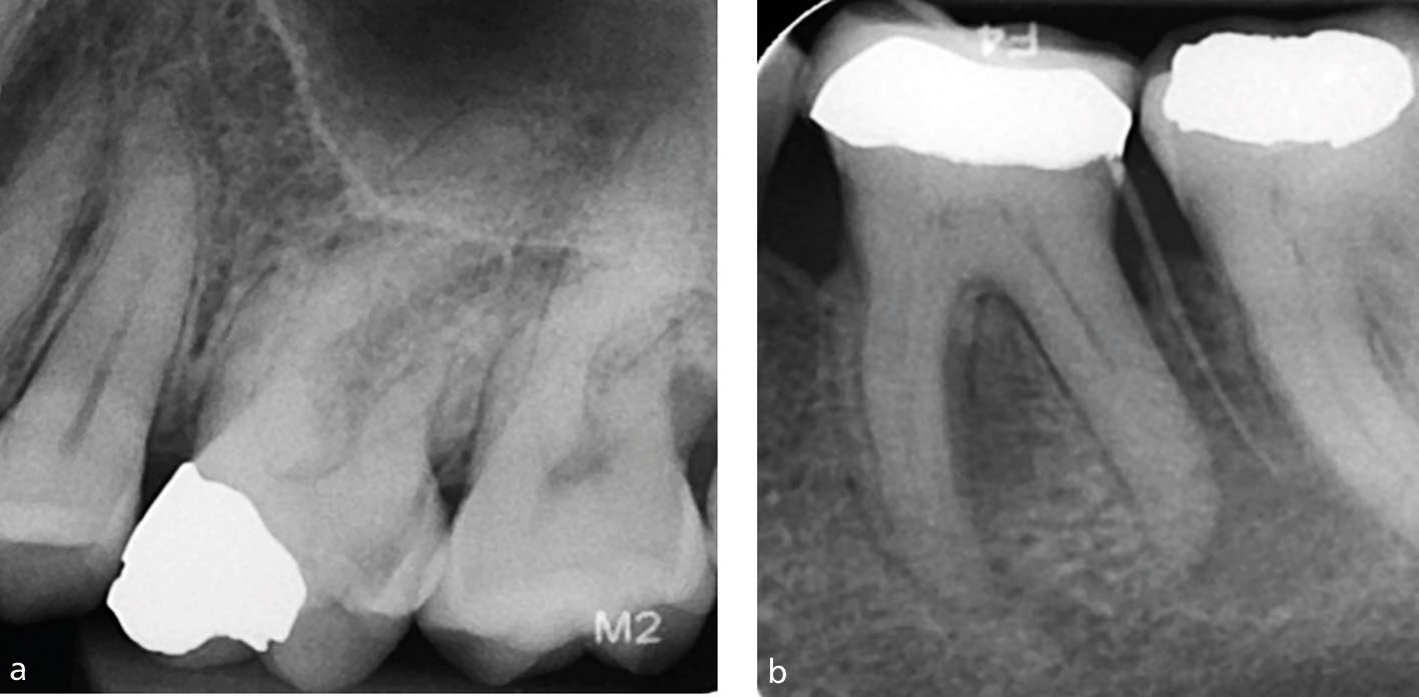
a) The 26 requiring root canal treatment, with a significantly reduced pulp chamber, indicating higher complexity. b) The 36 with a reduced pulp chamber, also indicating additional complexity. Note the gutta-percha cone showing the location that the sinus tract is originating from. The reduced sized pulp chambers increase the complexity of treatment due to increased risk of iatrogenic damage including perforation

As part of the pre-operative assessment, radiographic assessment is crucial in determining the size and height of the pulp chamber. Cases with a large pulp chamber present radiographically, such as those shown in Figure 5, will often carry less complexity, as clinicians will likely feel a ‘drop' when accessing these teeth with their bur of choice, thus reducing the risk of complications.

**Fig. 6:**
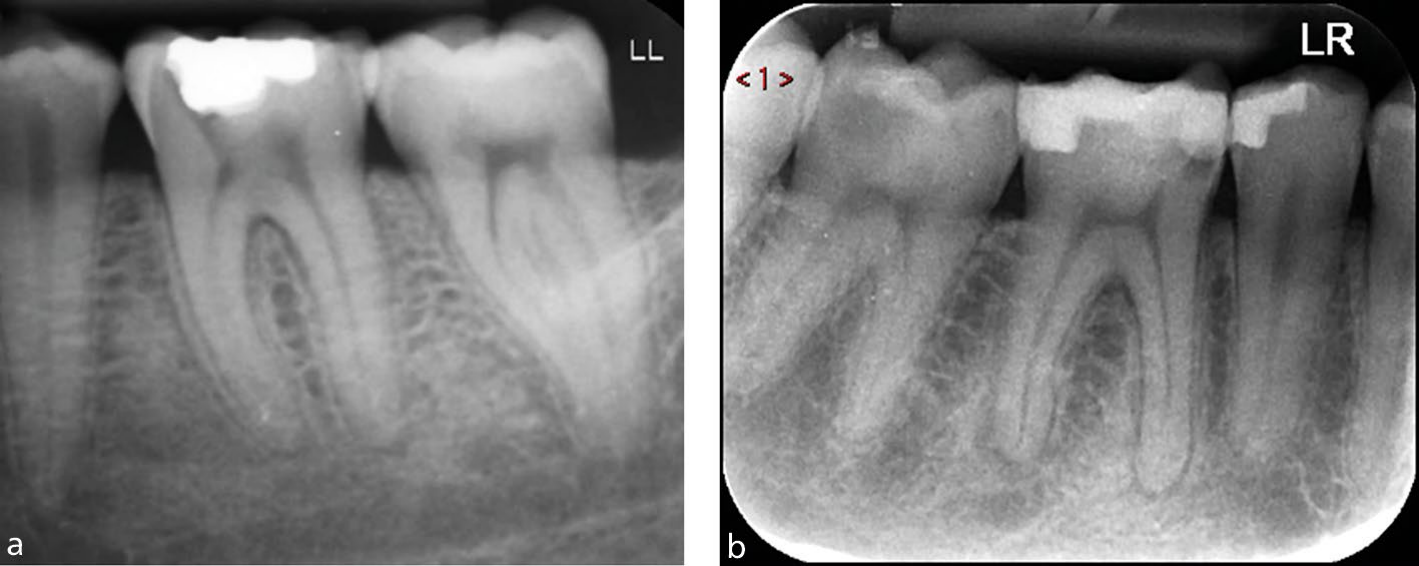
a, b) Periapical radiographs showing the 36 and 46, in both cases requiring endodontic treatment, with large, visible pulp chambers. This reduces the complexity of access cavity preparation, as the pulp chamber and canal orifices will be more easily located

However, other teeth may present with smaller and reduced pulp chambers, such as those shown in Figure 6, and these can carry higher complexity, as clinicians are unlikely to feel a ‘drop' when accessing these cases, and this may be due to a reduced/invisible pulp chamber, or due to calcifications such as pulp stones.

It is often good practice to use a measuring tool using the radiographic viewing software to measure the distance from the top of the crown to the roof of the pulp chamber on periapical radiographs, and additionally from the top of the crown to the cemento-enamel junction. Krasner and Rankow (2004) advise that the cemento-enamel junction is the most consistent repeatable landmark for locating the positions of the pulp chamber.^16^ Although the measurement taken radiographically can be influenced by foreshortening or elongation, a bitewing radiograph can give a more accurate reading in this regard.

Use of good lighting and magnification will aid identification of the canal orifice(s), developmental fusion lines and the ‘colour change' often mentioned when identifying the floor of the pulp chamber.^16^ The choice of initial bur when making the initial ‘pilot hole' into the pulp chamber is important, and it is the opinion of the authors that a round diamond or cylindrical bur, such as a 541 or a tapered 556, be used for this purpose. Whatever the choice, the operator needs to be aware of the length of the cutting diamonds and stop and re-assess the angle and orientation regularly, and most importantly, stop if the end of these is reached. A longer crown preparation bur (>8 mm) should be avoided due to the risk of perforation. Once the pulp chamber has been uncovered, the pulp chamber can be deroofed in its entirety with the use of a non-end-cutting tungsten carbide bur, such as an Endo-Z bur (Dentsply Sirona, USA); diamond equivalents are also available. The use of a non-end-cutting bur reduces the risk of damage to the pulp chamber floor, reducing the risk of perforation. It is imperative to keep the non-end-cutting bur parallel to the long axis of the tooth to reduce the risk of excessive tooth tissue removal. Alternatively, a slow-speed, rose-head, stainless steel bur can also be used to deroof the pulp chamber. At various intervals, a ball-ended or Briault probe can be used to explore the walls of the current access cavity to identify any undercuts indicating remaining pulp chamber roof.

Krasner and Rankow's laws also provide guidance on identifying the floor of the pulp chamber and canal orifices. The floor of the pulp chamber is normally a darker colour than the surrounding dentine walls, and the canal orifices are always located at the junction between the walls and floor of the pulp chamber.^16^ Darker developmental root fusion lines exist which can map out the pulp chamber floor, and the canal orifices are at the end of these fusion lines. Pulp stones can also affect identification of the pulp chamber floor and canal orifices and these can be identified pre-operatively on the radiograph through calcifications/radiopacities seen within the pulp chamber. Clinically, pulp stones often appear to carry a more ‘glassy' appearance as compared to the surrounding dentine.

Piezoelectric ultrasonics are very effective at removing pulp stones, while standard tips are often adequate. Thinner tips, such as diamond-coated Acteon ET-18D (Acteon, UK) or E15D (NSK, Japan) or grooved profile Start-X series (Dentsply Sirona, USA) are often the instrument of choice for clearing pulp stones. In cases such as those seen in Figure 6, due to the lack of a ‘drop' felt when accessing these teeth, care must be exercised and it is important that slow-speed burs, such as a Muller pulp or Gooseneck bur, be used, along with ultrasonics and plenty of irrigation and patience, in order to locate the canal orifices. A DG-16 endodontic explorer can then be used to identify the canal orifices, followed by scouting of the canal(s).

### Minimally invasive access cavity

The minimally invasive access cavity (MIAC) preparation aims to preserve tooth integrity by removing only necessary tooth structure required for instrumentation and disinfection. This method differs from traditional access cavity preparation, which involves the removal of a larger volume of tooth structure beyond that of the pulp chamber. Advances in technology, including the use of flexible instruments, magnification, and 3D-imaging techniques, such as cone beam computed tomography (CBCT), have made dentine conservation a feasible goal.^17^ This is particularly important in the peri-cervical region of the tooth, where the pulp chamber meets root canal, in the distribution of occlusal stress and the prevention of cracked teeth.^18^

Over the last 20 years, our understanding of root canal anatomy and its variations have developed significantly and have gone hand-in-hand with the wider acceptance and use of 3D-imaging modalities, along with *in vitro* micro-computed tomography studies of extracted human teeth.^19^ While these studies have confirmed what was already known about the gross anatomy of the pulp chamber, with CBCT, there is the ability to visualise canal orifices, complex branching and anastomoses, and the presence or absence of ‘additional' canals before access preparation.^20^

MIAC involves a thorough pre-operative radiographic identification of the root canal system, including its variations, and the presence of calcifications. With this information, the access cavity is meticulously designed. The access cavity is then guided with precision using dental magnification, along with small instruments with long shanks, such as ultrasonic or Muller pulp bur instruments.

**Fig. 7:**
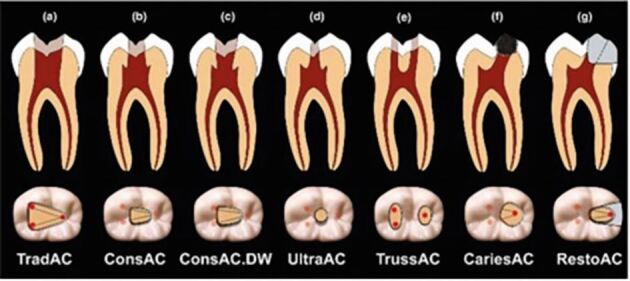
Showing different types of access cavities. (TradAC = traditional; ConsAC = conservative; UltraAC = ultraconservative; TrussAC = truss; CariesAC = caries-orientated; RestoAC = restorative). Reproduced with permission from Silva *et al.*, ‘Current status on minimal access cavity preparations: a critical analysis and a proposal for a universal nomenclature', *International Endodontic Journal*, 2020, Wiley

There are several techniques to achieve an MIAC, as outlined below and shown in Figure 7:

- Traditional access cavity
- Conservative access cavity - note the convergent access with partial removal of pulp chamber roof
- Conservative access cavity with divergent walls
- Ultra-conservative access cavity (ninja access) - this approach is similar to the conservative access cavity but with no additional extensions beyond the initial opening
- Truss access cavity - this technique involves creating separate access cavities on the occlusal surface to expose the canal orifices while preserving the middle dentine
- Caries-driven access cavity - access to the pulp chamber is achieved by removing caries while preserving all remaining sound dentine
- Restorative-driven access cavity - access to the pulp chamber is achieved by partially or fully removing the restorations while preserving as much of the remaining tooth structure.

Preserving more sound tooth structure when preparing MIAC is thought to reduce the risk of tooth fracture.^21^ However, MIAC comes with risks and challenges and requires a high level of operator skill and specialised equipment, such as magnification and ultrasonic instruments. A major limitation is the difficulty in adequately mapping the pulp floor to identify canal orifices, particularly in multirooted teeth. This approach needs direct visual control, making the use of high-speed burs impractical, since the handpiece head often obstructs the view during procedures. In this scenario, ultrasonic tips with longer shanks (Fig. 1) have become a basic technical requirement for performing such methods.^22^ As a result, it can be more time-consuming and lead to increased fatigue for both patients and clinicians.^23^^,^^24^^,^^25^ As these are relatively new techniques, there are currently no robust, long-term, tooth survival outcome studies to suggest that MIAC root canal-treated teeth have a better survival outcome than those with traditional access cavity preparation.^26^

Such minimal accesses can lead to risks such as:

- Missed canals and incomplete cleaning of the entire pulp chamber and root canals
- Postoperative pain and sensitivity due to incomplete removal of infected tissue
- Instrument fracture within the canal due to increased stress on files
- The remaining tooth structure can still be prone to postoperative fracture if not adequately supported by appropriate restorative materials.

### CBCT-informed and guided access

**Fig. 8:**
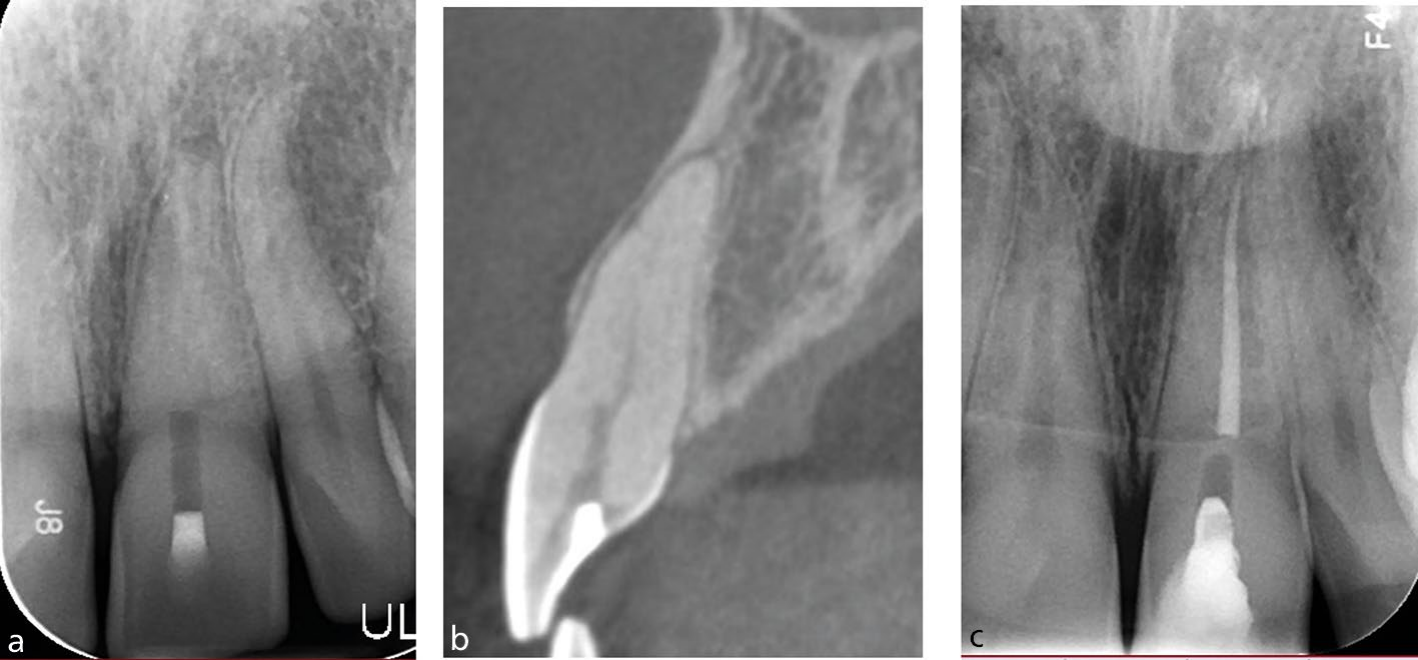
a) Pre-operative periapical radiograph of the 21, with an attempted access cavity by the patient's general dental practitioner. b) Sagittal CBCT slice showing a clear and evident canal emanating from the palatal aspect of the current access cavity. c) Periapical radiograph showing completed root canal treatment for the 21, with the gutta-percha sealed with resin-modified glass ionomer cement, and the 21 now ready for internal/external bleaching. The canal was located using high magnification and the CBCT

In teeth with reduced pulp chambers and calcified canals, CBCT can be an invaluable tool for canal location in various planes. A modern, ‘small field of view' CBCT carries a low radiation dose, meaning the benefits will outweigh the risks in many cases.^27^ Figure 8 shows the 21 which had been referred by the patient's general dental practitioner, who had been unable to locate the canal. The 21 had undergone pulp canal obliteration because of a previous traumatic injury. The pre-operative periapical radiograph shows an attempted access cavity with an indistinct/invisible canal evident.

A CBCT was taken to assess canal location and this revealed a clear, distinct canal which was unclear on the 2D radiograph (Fig. 8a). As the location of the canal in relation to the existing access cavity could now be easily determined, the canal was easily located within minutes using high magnification and the CBCT, with a minimal loss of additional tooth structure. This illustrates the value that CBCT has, especially when used following an attempted access, as the access cavity can then be re-oriented in the correct direction. Additionally, CBCT can reveal a distinct canal when one is not visible in conventional 2D radiography, and a measuring tool on the CBCT viewing software can dictate the depth of the access cavity with complete accuracy.

In conjunction with the above, ‘guided' endodontics can also be an invaluable tool for locating calcified canals. The use of a static guide is reported below to aid canal location; although, dynamic navigation has also been discussed in case reports in the literature.^28^ The construction of a guide for access of a calcified canal begins with a CBCT and intra-oral scan/digitised impression of the arch. These are then transferred into implant planning software, such as coDiagnostiX (coDiagnostiX, Germany), where both the CBCT scan and intra-oral scan are overlayed. Virtual planning of the angulation and orientation of the model can take place so that the tip of the bur corresponds with both the start of the visible canal on the CBCT while remaining within the long axis of the tooth. From this, a stent can then be designed and 3D-printed, which seats on the tooth of interest and neighbouring teeth with minimal offset. A metal sleeve with a diameter of 1 mm is then placed into the stent, which the titanium drill can be placed through.

**Fig. 9:**
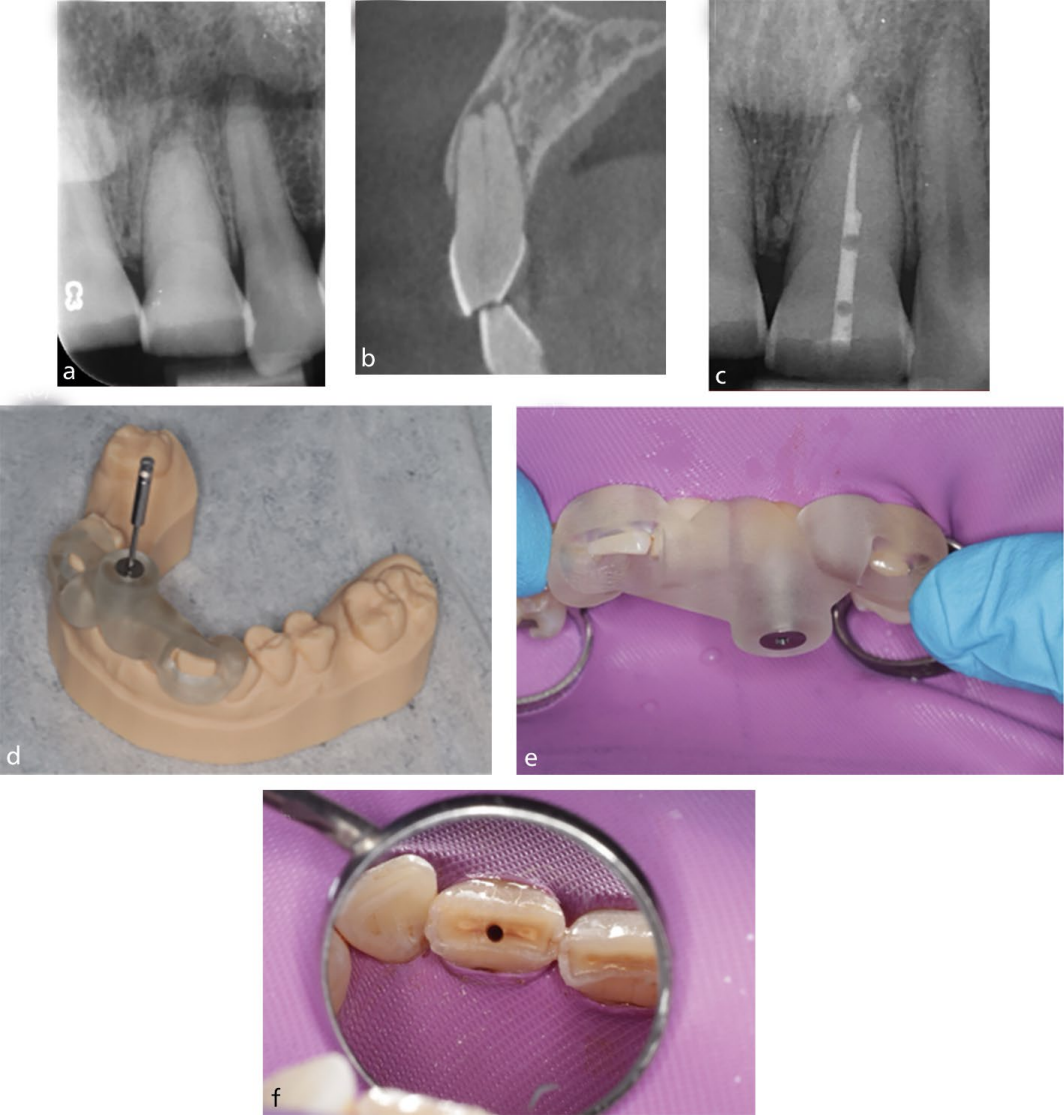
a) Periapical radiograph of the 21 with evidence of apical pathology and an invisible canal. b) Sagittal CBCT slide showing evidence of a patent canal with associated apical pathology around the 21. c) Postoperative periapical radiograph following root canal treatment of the 21. Note the voids in the access cavity restored with resin composite, owing to the very conservative nature of the access cavity. d) 3D-printed stent seating fully on model. Note the viewing windows designed to check for full seating of the guide. e) Full seating of the stent, verified through the viewing windows added. f) Access cavity created by the Steco titanium drill

The following case demonstrates the successful use of a guide to aid canal location. A male patient in their fifties was referred regarding the 21, which had evidence of pulp canal obliteration with an associated area of apical pathology (Fig. 9a).^29^ A CBCT was taken which revealed evidence of canal space from the coronal third of the root (Fig. 9b).

An intra-oral scan was then taken and coDiagnostiX was used to design a stent (Fig. 9c), where the tip of the titanium drill (Fig. 9d) was virtually simulated to access the canal orifice parallel with the long axis of the tooth. Once the stent was finalised, and following appropriate anaesthesia and dental dam isolation, the stent was fully seated and a Steco titanium 1 mm diameter drill (Steco-System-Yechnik, Germany) was used in a slow handpiece at 10,000 RPM through the sleeve in short pumping motions, until the canal could be scouted with a small hand file.

Guided access using a static guide does have limitations. It can only be used in roots with no/minor curvatures and can lead to incisal/slightly labially placed access cavities, which can have aesthetic implications. It can also be difficult to use this technique when the tooth in question or adjacent teeth are heavily restored, owing to scatter caused by restorations on the CBCT scan. Using a static guide on posterior teeth can also be difficult, owing to the patient's mouth opening impacting stent seating and use of the drill. Furthermore, many experienced clinicians will be able to locate calcified canals without the use of the stent; however, the use of the guide can aid clinicians of varying levels of experience to locate canals efficiently and predictably. Additionally, this method results in the loss of less-sound tooth tissue as compared to conventional techniques and reduces the risk of perforation. Figure 9f shows the size of the access cavity created by the Steco drill and Fig. 9c shows the postoperative radiographh.

## Conclusion

Access cavity preparation can be challenging for all clinicians, particularly in teeth with higher levels of complexity. Dental, operator and patient factors can affect predictability of endodontic access. Moreover, the operator's skill and experience and the use of correct equipment and magnification are paramount. Patient factors can include the level of cooperation and mouth opening and should be assessed as part of the pre-operative assessment.

Case complexity can be determined using tools such as the E-CAT (Endodontic Complexity Assessment Tool) and cases that carry higher complexity should be referred appropriately. Clinicians undertaking root canal treatment should carry out an initial pre-operative assessment using CBCT when needed, which can be an invaluable tool to orientate clinicians in three dimensions when difficulties are encountered.^30^

## References

[1] Edwards D, Rasaiah S, Hamzah Ahmed S *et al*. The financial and quality of life impact of urgent dental presentations: a cross-sectional study. *Int Endod J* 2023; **56:** 697-709. 10.1111/iej.13917 36975836

[2] Duncan H F, Kirkevang L L, Peters O A *et al*. Treatment of pulpal and apical disease: the European Society of Endodontology (ESE) S3-level clinical practice guideline. *Int Endod J* 2023; **56:** 238-295. 10.1111/iej.13974 37772327

[3] Edwards D, Stone S, Bailey O, Tomson P. Preserving pulp vitality: part two-vital pulp therapies. *Br Dent J* 2021; **230:** 148-155. 10.1038/s41415-020-2599-y 33574536

[4] Wolters W J, Duncan H F, Tomson P L *et al*. Minimally invasive endodontics: a new diagnostic system for assessing pulpitis and subsequent treatment needs. *Int Endod J* 2017; **50:** 825-829. 10.1111/iej.12793 28776717

[5] Fouad A F, Abbott P V, Tsilingaridis G *et al*. International Association of Dental Traumatology guidelines for the management of traumatic dental injuries: 2. Avulsion of permanent teeth. *Dent Traumatol* 2020; **36:** 331-342. 10.1111/edt.12573 32460393

[6] European Society of Endodontology. Quality guidelines for endodontic treatment: consensus report of the European Society of Endodontology. *Int Endod J* 2006; **39:** 921-930. 10.1111/j.1365-2591.2006.01180.x 17180780

[7] Beach C W, Calhoun J C, Bramwell J D, Hutter J W, Miller G A. Clinical evaluation of bacterial leakage of endodontic temporary filling materials. *J Endod* 1996; **22:** 459-462. 10.1016/S0099-2399(96)80077-X 9198425

[8] Krakow A A, de Stoppelaar J D, Grøn P. *In vivo* study of temporary filling materials used in endodontics in anterior teeth. *Oral Surg Oral Med Oral Pathol* 1977; **43:** 615-620. 10.1016/0030-4220(77)90117-7 265490

[9] Abbott P V. Assessing restored teeth with pulp and periapical diseases for the presence of cracks, caries and marginal breakdown. *Aust Dent J* 2004; **49:** 33-39. 10.1111/j.1834-7819.2004.tb00047.x 15104132

[10] Sorensen J A, Engelman M J. Ferrule design and fracture resistance of endodontically treated teeth. *J Prosthet Dent* 1990; **63:** 529-536. 10.1016/0022-3913(90)90070-s 2187080

[11] British Endodontic Society. A guide to good endodontic practice. 2022. Available at https://britishendodonticsociety.org.uk/news/39/a_guide_to_good_endodontic_practice/ (accessed March 2025).

[12] British Endodontic Society. BES case assessment tool. 2018. Available at https://britishendodonticsociety.org.uk/professionals/bes_case_assessment_tool.aspx (accessed September 2024).

[13] Virdee S S, Thomas M B M. A practitioner's guide to gutta-percha removal during endodontic retreatment. *Br Dent J* 2017; **222:** 251-257. 10.1038/sj.bdj.2017.166 28232689

[14] American Association of Endodontists. AAE endodontic case difficulty assessment form and guidelines. 2006. Available at https://www.aae.org/specialty/wp-content/uploads/sites/2/2022/01/CaseDifficultyAssessmentFormFINAL2022.pdf (accessed March 2025).

[15] Essam O, Boyle E L, Whitworth J M, Jarad F D. The Endodontic Complexity Assessment Tool (E-CAT): a digital form for assessing root canal treatment case difficulty. *Int Endod J* 2021; **54:** 1189-1199. 10.1111/iej.13506 33682086

[16] Krasner P, Rankow H J. Anatomy of the pulp-chamber floor. *J Endod* 2004; **30:** 5-16. 10.1097/00004770-200401000-00002 14760900

[17] Bóveda C, Kishen A. Contracted endodontic cavities: the foundation for less invasive alternatives in the management of apical periodontitis. *Endod Top* 2015; **33:** 169-186.

[18] Krishan R, Paqué F, Ossareh A, Kishen A, Dao T, Friedman S. Impacts of conservative endodontic cavity on root canal instrumentation efficacy and resistance to fracture assessed in incisors, premolars, and molars. *J Endod* 2014; **40:** 1160-1166. 10.1016/j.joen.2013.12.012 25069925

[19] Ahmed H M A, Ibrahim N, Mohamad N S *et al*. Application of a new system for classifying root and canal anatomy in studies involving micro-computed tomography and cone beam computed tomography: Explanation and elaboration. *Int Endod J* 2021; **54:** 1056-1082. 10.1111/iej.13486 33527452

[20] Bürklein S, Schäfer E. Minimally invasive endodontics. *Quintessence Int* 2015; **46:** 119-124. 10.3290/j.qi.a33047 25500587

[21] Clark D, Khademi J. Modern molar endodontic access and directed dentin conservation. *Dent Clin North Am* 2010; **54:** 249-273. 10.1016/j.cden.2010.01.001 20433977

[22] Plotino G, Pameijer C H, Grande N M, Somma F. Ultrasonics in endodontics: a review of the literature. *J Endod* 2007; **33:** 81-95. 10.1016/j.joen.2006.10.008 17258622

[23] Van der Sluis L W, Versluis M, Wu M K, Wesselink P R. Passive ultrasonic irrigation of the root canal: a review of the literature. *Int Endod J* 2007; **40:** 415-426. 10.1111/j.1365-2591.2007.01243.x 17442017

[24] Boutsioukis C, Arias-Moliz M T. Present status and future directions - irrigants and irrigation methods. *Int Endod J* 2022; **55:** 588-612. 10.1111/iej.13739 PMC9321999 35338652

[25] Peters O A, Bardsley S, Fong J, Pandher G, Divito E. Disinfection of root canals with photon-initiated photoacoustic streaming. *J Endod* 2011; **37:** 1008-1012. 10.1016/j.joen.2011.03.016 21689561

[26] Al-Helou N, Zaki A A, Al Agha M, Moawad E, Jarad F. Which endodontic access cavity is best? A literature review. *Br Dent J* 2023; **234:** 335-339. 10.1038/s41415-023-5581-7 36899249

[27] Chan F, Brown L F, Parashos P. CBCT in contemporary endodontics. *Aust Dent J* 2023; **68:** 39-55. 10.1111/adj.12995 37975281

[28] Connert T, Weiger R, Krastl G. Present status and future directions - guided endodontics. *Int Endod J* 2022; **55:** 995-1002. 10.1111/iej.13687 PMC9790195 35075661

[29] Robertson A, Andreasen F M, Bergenholtz G, Andreasen J O, Norén J G. Incidence of pulp necrosis subsequent to pulp canal obliteration from trauma of permanent incisors. *J Endod* 1996; **22:** 557-560. 10.1016/S0099-2399(96)80018-5 9198446

[30] Patel S, Brown J, Semper M, Abella F, Mannocci F. European Society of Endodontology position statement: use of cone beam computed tomography in Endodontics: European Society of Endodontology (ESE) developed by. *Int Endod J* 2019; **52:** 1675-1678. 10.1111/iej.13187 31301231

